# Dynamic brain network reconfiguration following rTMS in males with cocaine use disorder

**DOI:** 10.3389/fnhum.2025.1603888

**Published:** 2025-08-21

**Authors:** Chengjie Le, Tianyu Hou, Yifan Zhu, Yaoyin Zhang, Jianjie Ling, Zixian Li, Benteng Sun, Ting Ma, Chenfei Ye

**Affiliations:** ^1^School of Robotics and Advanced Manufacturing, Harbin Institute of Technology (Shenzhen), Shenzhen, China; ^2^Department of Electronic and Information Engineering, Harbin Institute of Technology (Shenzhen), Shenzhen, China; ^3^School of Economics and Management, Harbin Institute of Technology (Shenzhen), Shenzhen, China; ^4^School of Science, Harbin Institute of Technology (Shenzhen), Shenzhen, China; ^5^School of Biomedical Engineering, Harbin Institute of Technology (Shenzhen), Shenzhen, China; ^6^Peng Cheng Laboratory, Shenzhen, China

**Keywords:** brain state, cocaine use disorder, resting-state fMRI, rTMS, LEiDA

## Abstract

Cocaine use disorder (CUD) is characterized by cortico-striatal circuit dysregulation and high relapse rates, with repetitive transcranial magnetic stimulation (rTMS) emerging as a potential neuromodulatory intervention. This study investigates rTMS-induced dynamic brain network reconfigurations in 30 CUD patients using longitudinal resting-state fMRI from the SUDMEX-TMS cohort. Applying Leading Eigenvector Dynamics Analysis (LEiDA) to phase-locking states, we identified four metastable network configurations mapped to canonical resting-state networks. Post-rTMS analyses revealed selective modulation of visual network (VIS)-dominant states, showing increased duration and occupancy, alongside reduced self-transition probabilities in frontoparietal control network (FPCN) states after rTMS therapy. Temporal dynamics of these states correlated with subjective craving intensity: increased duration of the VIS-dominant state was associated with lower craving severity (CCQ-N) post-treatment. These findings suggest that increased VIS metastability strengthened bottom-up sensory gating that attenuates drug-cue salience through perceptual desensitization. Although FPCN-state self-transition decreased significantly following stimulation, it was not directly linked to craving improvement, indicating a potentially supportive but nonspecific role in perceptual recalibration. Together, these dynamic markers highlight the relevance of network-level flexibility in mediating rTMS treatment efficacy for cocaine addiction. By establishing dynamic network state reconfiguration as a mechanism linking rTMS to symptom evolution, this work provides a framework for optimizing neuromodulation protocols and developing neurodynamics-dependent biomarkers in addiction therapeutics.

## Introduction

Cocaine use disorder (CUD) is a severe neuropsychiatric condition characterized by persistent cocaine-seeking behavior despite adverse consequences, reflecting dysregulation of cortico-striatal reward circuits (Huang et al., 2017). While psychosocial interventions provide partial symptom relief, high relapse rates persist due to limited interventions targeting the neurobiological substrates of addiction (Poireau et al., 2022). Repetitive transcranial magnetic stimulation (rTMS), a non-invasive neuromodulation technique based on electromagnetic induction principles, offers potential normalizing addiction-related neural dysfunction by targeting prefrontal cortical regions (Shen and Ward, 2021).

Preclinical and clinical studies demonstrate that high-frequency rTMS over the dorsolateral prefrontal cortex (dlPFC) can reduce cocaine craving and modulate dopamine-dependent reward processing (Camprodon et al., 2007; Moretti et al., 2020). Theta burst stimulation protocols further show comparable efficacy to conventional rTMS in attenuating addictive behaviors, potentially through restoring inhibitory control over limbic-striatal systems (Sanna et al., 2019). Additionally, rTMS-induced improvements in comorbid sleep disturbances and affective symptoms suggest broader network-level effects beyond primary addiction circuits (Gómez Pérez et al., 2020; Martinotti et al., 2022). However, Lolli et al. (2021) reported substantial variability in treatment outcomes among individuals receiving rTMS. In their randomized controlled trial, only a subset of participants achieved sustained abstinence, suggesting that a significant proportion of patients may show limited clinical response to the intervention. This heterogeneity underscores two critical knowledge gaps: (1) incomplete characterization of rTMS-mediated neural plasticity in CUD, and (2) lack of biomarkers predicting intervention efficacy.

A fundamental limitation in current research lies in reliance on static functional connectivity (FC) measures, which fail to capture dynamic network reconfigurations central to addictive behaviors (Hutchison et al., 2013). One major limitation of static functional connectivity (FC) analysis is its assumption of temporal stationarity across the entire fMRI scanning session. This approach typically computes correlations between brain regions over several minutes, thereby overlooking the inherently dynamic nature of neural activity. As a result, important transient fluctuations in connectivity, which may reflect meaningful cognitive or pathological states, are effectively averaged out and lost. Additionally, static FC provides only undirected, averaged correlation patterns and does not capture the temporal ordering or directionality of interactions between brain regions, limiting its utility in understanding the mechanisms of brain network organization (Hutchison et al., 2013). These shortcomings have prompted growing interest in dynamic FC approaches that better account for the time-varying nature of brain connectivity. Dynamic functional connectivity (dFC) captures time-varying patterns of brain network interactions and is more sensitive to transient neural changes than static FC. Given that rTMS induces temporally evolving neuroplastic effects, dynamic FC provides a suitable framework to track these changes. The Leading Eigenvector Dynamics Analysis (LEiDA) framework addresses this gap by quantifying transient brain states through phase-locking (PL) patterns in resting-state fMRI (rs-fMRI) signals (Alonso et al., 2023). This method identifies recurring functional network configurations (dwell time) and transition probabilities between states—metrics particularly relevant for addiction phenotypes characterized by impaired cognitive flexibility (Wang et al., 2023). Unlike traditional static FC or dynamic approaches such as sliding-window correlation, LEiDA avoids arbitrary windowing and instead extracts the leading eigenvector of the BOLD phase coherence matrix at each time point, yielding a compact and time-resolved representation of whole-brain connectivity. This approach is particularly advantageous for its high temporal sensitivity, robustness to noise, and ability to identify recurrent connectivity states relevant to behavior or clinical outcomes (de Alteriis et al., 2024). These features make LEiDA well-suited for detecting subtle, fast-evolving network reconfigurations associated with rTMS, and for exploring potential biomarkers of treatment efficacy. Although LEiDA has revealed altered metastable dynamics in substance use disorders, its application to understand rTMS-induced neural changes remains unexplored (Zheng et al., 2025).

The efficacy of rTMS in addiction treatment is critically influenced by stimulation target localization. While novel targeting approaches are emerging, the left dorsolateral prefrontal cortex (dlPFC) remains the most empirically validated target for addictive disorders (Kazemi et al., 2018; Liston et al., 2014). In this study, we do not evaluate target placement per se, but capitalize on a standardized rTMS treatment paradigm—where all participants received dlLPFC stimulation—to address a distinct gap: how inter-individual variability in intrinsic brain dynamics predicts response to an otherwise fixed intervention protocol.

Building on this established protocol, the present study leverages longitudinal fMRI data from 30 selected male individuals with CUD undergoing rTMS treatment to (1) characterize dynamic FC alterations before and after intervention, and (2) map relationships between network dynamic reconfiguration and clinical outcomes. By applying LEiDA to track rTMS-mediated changes in metastable brain states, we establish a computational framework for understanding neuromodulation mechanisms in addiction. Our findings demonstrate dlPFC-targeted rTMS stimulation preferentially enhancing activity in the sensorimotor network (SMN), regulating the visual network (VIS), and suppressing self-directed rumination associated with the dynamics of the frontoparietal control network (FPCN), providing critical insights for optimizing therapeutic protocols.

## Methods

**Figure 1:**
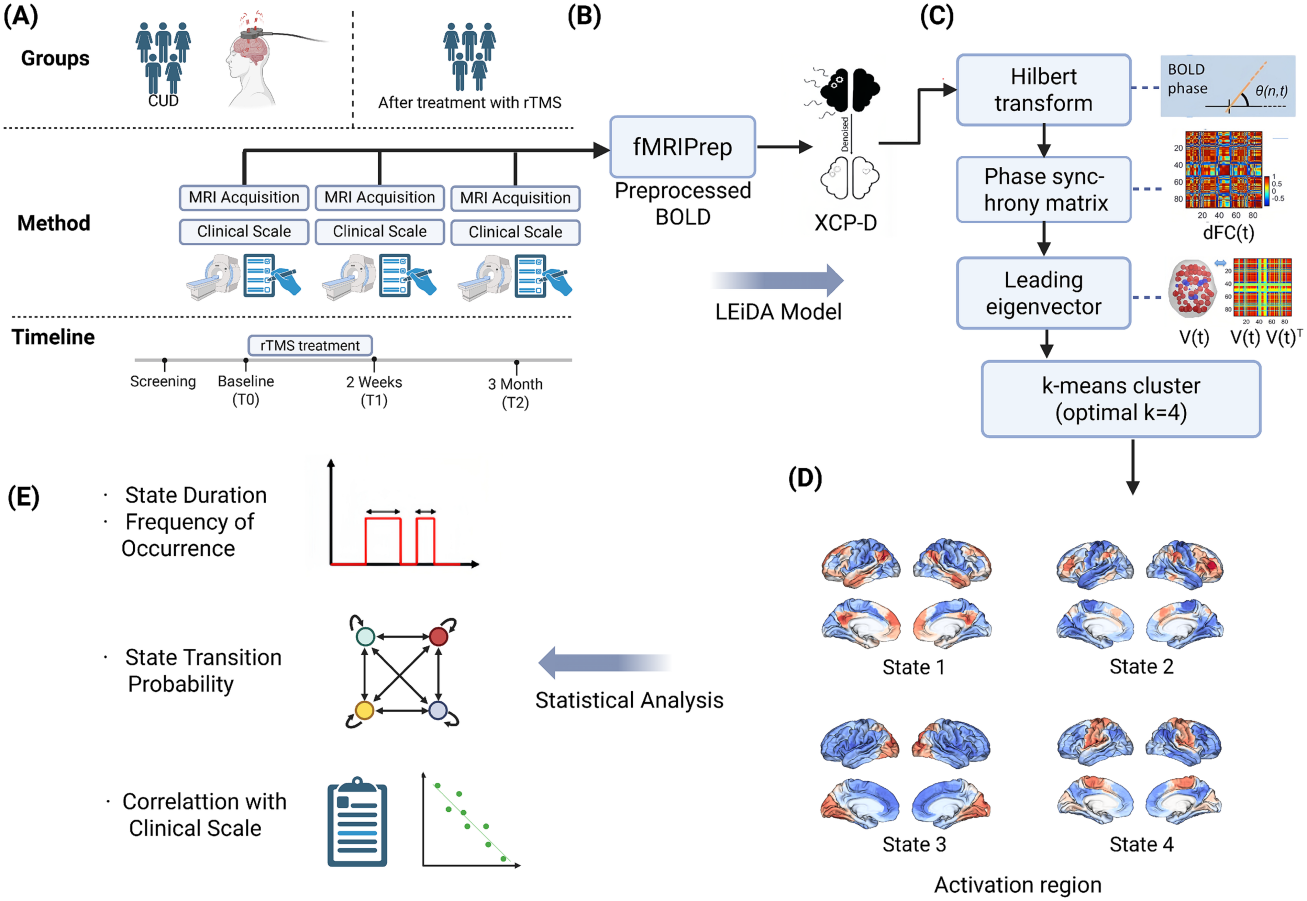
The schematic illustrations of the study methodology. **(A)** Neuroimaging data were collected from the SUDMEX-TMS multimodal neuroimaging initiative, including resting-state fMRI scans at baseline, post-rTMS, and two follow-up stages. **(B)** Data were preprocessed using fMRIPrep and XCP-D pipelines. **(C)** The BOLD signals extracted from each brain region were first transformed into instantaneous phase time series using the Hilbert transform. At each time point, a phase synchrony matrix was computed. From each matrix, the leading eigenvector was extracted to summarize the dominant pattern of phase synchrony at that moment, yielding a time series of principal connectivity modes. These eigenvectors were then input into a k-means clustering algorithm. Based on multiple clustering validity indices, the optimal number of states was determined as *k* = 4. **(D)** The spatial maps of each state were projected onto Yeo’s 7-network atlas, revealing distinct large-scale connectivity patterns anchored in canonical networks. **(E)** We quantified several temporal features of brain state dynamics: (1) fractional occupancy (duration), (2) frequency of occurrence, (3) transition probability matrix, and further examined their longitudinal changes across sessions. These dynamic metrics were then statistically correlated with clinical outcomes (e.g., craving, impulsivity scores) to explore potential biomarkers of rTMS efficacy.

Our analytical pipeline systematically addressed five critical phases of dynamic network characterization: (a) neuroimaging data acquisition from SUDMEX-TMS multimodal neuroimaging initiative (Angeles-Valdez et al., 2024); (b) preprocessing and LEiDA modeling; (c) optimal state number determination via elbow method inflection point; (d) Spatial projection of cluster centroids onto Yeo’s 7-network template (Yeo et al., 2011), revealing distinct configurations spanning canonical resting-state networks; (e) temporal feature quantification and statistical analysis. See Figure 1 for details.

### Study participants

The discovery cohort comprised individuals with cocaine use disorder (CUD) recruited from the Clinical Research Division of the National Institute of Psychiatry in Mexico City, Mexico, as part of the SUDMEX-TMS multimodal neuroimaging initiative (Angeles-Valdez et al., 2024). Fifty-four treatment-seeking participants meeting DSM-5 criteria for CUD were enrolled in a longitudinal rTMS intervention protocol. Inclusion criteria required: (1) above 1 year of documented cocaine use, (2) average use frequency longer than 3 days/week during the preceding 12 months, and (3) abstinence duration less than 1 month in the past year. Exclusion criteria encompassed major neurological/neurodegenerative disorders, contraindications for MRI/rTMS (e.g., metallic implants), and active comorbid psychiatric conditions requiring hospitalization. All participants underwent structured clinical interviews conducted by board-certified psychiatrists to verify eligibility. Prior to enrollment, detailed study protocols, including safety considerations, potential adverse effects, and data anonymization procedures, were disclosed during informed consent sessions supervised by an independent ethics committee. For rTMS therapy targeting on the left dlPFC, MagPro R301 stimulator with figure-of-eight B65-A/P coil (MagVenture) was employed, with the following parameters: 5 Hz frequency, 100% resting motor threshold (MT) intensity (determined via Rossini method), 5,000 pulses/day (two daily sessions: 50 trains/session, 50 pulses/train, 10 s inter-train interval, 15 min inter-session interval).

Male participants were exclusively analyzed in this study due to the limited number of female participants (*n* = 8) and the potential confounding influence of sex as a biological variable. The two original cohorts of male participants receiving active rTMS treatment were consequently merged, yielding a final experimental group of 30 participants to enhance statistical power. At the baseline (T0) session, participants underwent a full clinical evaluation and an initial MRI scan before receiving any rTMS intervention. During the subsequent acute stage (RCT), participants received 10 sessions of rTMS over two consecutive weeks. Immediately following this two-week intervention, participants underwent a second clinical evaluation and MRI scan at Time 1 (T1), allowing assessment of neural changes associated with the initial rTMS treatment.

Participants underwent an acute phase consisting of 10 sessions delivered over 2 weeks (post-rTMS-2 W), followed by a maintenance phase comprising twice-weekly sessions for up to 3 months (post-rTMS-3 M). The maintenance phase included up to 26 sessions, bringing the total number of rTMS sessions to a maximum of 36 per participant (10 acute + 26 maintenance).

### Clinical assessment

Cocaine use disorder diagnosis was confirmed using the Spanish-language MINI International Neuropsychiatric Interview (version 5.0.0) administered by certified clinicians (Sheehan et al., 1998). Comprehensive demographic profiling captured age, educational attainment (years), and socioeconomic status (monthly income in Mexican pesos, MXN). Cocaine use patterns were systematically documented through self-reported measures of consumption duration, past treatment history, onset age of cocaine use and route of administration. The demographic and clinical characteristics of all participants are demonstrated in Tables 1, 2, respectively. Four standardized instruments quantified addiction-related symptomatology: the Visual Analogue Scale (VAS) capturing momentary craving intensity (0–100 mm scale), the Cocaine Craving Questionnaire-General (CCQ-G) evaluating multidimensional craving traits (16 items, 7-point Likert scale), the Hamilton Anxiety Rating Scale (HARS) assessing somatic/psychic anxiety domains (14 items, 0–4 severity scoring), and the Cocaine Craving Questionnaire-Now (10 items, 7-point Likert scale). All scales were administered by trained psychiatrists at each assessment timepoint.

**Table 1:** Demographic characteristics of participants with CUD.

CUD participants (*n* = 30)	Baseline
Age	34.78 ± 7.13
Education (years)	13.12 ± 2.96
Monthly income (MXN)	5573.47 ± 8378.04
Crack cocaine as the main substance of use	28 (93.3%)
Onset age of cocaine use	22.12 ± 5.49
Years of cocaine use	11.38 ± 7.78
Received psychosocial treatment	*n* = 7 (23.3%)
Received pharmacological treatment	*n* = 24 (80.0%)

Continuous variables are reported as mean ± SD, and nominal as number (percentage from group).

**Table 2:** Clinical scales for CUD with statistical results using paired *t*-tests.

Clinical phenotypes	pre-rTMS (*n* = 30)	post-rTMS-2 W (*n* = 30)	*t*-value	*p*-value
VAS	3.5123 ± 3.5501	1.3700 ± 2.2932	4.5874	0.0001
CCQ-G	182.4667 ± 52.0919	147.6000 ± 50.2659	3.9598	0.0004
HARS	15.6667 ± 11.3117	8.0000 ± 8.1790	3.8079	0.0007
CCQ-N	146.8667 ± 48.9798	119.1333 ± 45.6997	4.0396	0.0004

VAS, Visual Analogue Scale; CCQ-G, General Craving Scale-General; HARS, Hamilton Anxiety Rating Scale; CCQ-N, General Craving Scale-Now.

### MRI scanning parameters

MRI sequences were acquired using a Philips Ingenia 3T MR system (Philips Healthcare, Best, The Netherlands, and Boston, MA, United States), with a 32-channel head coil. Each participant received MRI scanning at both the baseline period (pre-rTMS) and the follow-up period (post-rTMS). The scan of rs-fMRI session lasted 10 min, with 300 volumes acquired. Participants were instructed to keep their eyes open, relax, and avoid thinking about anything specific. MRI-compatible goggles displayed a fixation cross, and an eye-tracking camera was used to monitor participants and prevent sleep. Using a gradient recalled (GE) echo planar imaging (EPI) sequence, the rs-fMRI sequences were acquired: dummies = 5, repetition time (TR)/echo time (TE) = 2,000/30.001 ms, flip angle = 75°.matrix = 80 × 80, field of view = 240 mm^2^, voxel size = 3 × 3 × 3.33 mm, gap = 0, slice acquisition order = interleaved (ascending), number of slices = 36, phase encoding direction = AP.

### Data preprocessing and LEiDA analysis

Head motion was assessed using framewise displacement (FD). For each participant, we calculated the mean FD at T0 session. Participants with a mean FD greater than 0.5 mm were excluded, following commonly used criteria in the literature (Power et al., 2012). As a result, no male participants were excluded due to excessive head motion. All remaining data underwent motion correction and standard realignment procedures during preprocessing. Initial processing of rs-fMRI data was performed using fMRIprep, which leveraged individual high-resolution T1-weighted anatomical images 3D FFE SENSE sequence (Esteban et al., 2019); from SUDMEX-TMS dataset for robust anatomical-functional integration. This pipeline executed essential steps: T1 images were warped to the MNI152NLin6Asym standard space (6th generation); slice-time correction, rigid-body motion realignment, 6 mm FWHM Gaussian smoothing, and 0.01–0.1 Hz bandpass filtering (Angeles-Valdez et al., 2024). Subsequent denoising and quality control were conducted via XCP-D (Mehta et al., 2024), which executed: (1) nuisance regression (24 motion parameters, WM/CSF signals), (2) Schaefer 400-parcel atlas-based time series extraction, (3) functional connectivity matrix computation (Fisher-z transformed Pearson correlations), and (4) comprehensive quality assessment including framewise displacement and DVARS monitoring. The preprocessed BOLD signals underwent phase synchronization analysis through the following protocol: Time series from each Schaefer atlas-defined region were bandpass-filtered (0.01–0.1 Hz) to attenuate physiological noise while preserving neurovascular coupling-related oscillations. These filtered signals were then subjected to Hilbert transform for instantaneous phase estimation, enabling dynamic functional connectivity characterization. The Hilbert transform was applied to filtered BOLD signals to derive instantaneous phase estimates, enabling computation of pairwise phase differences across all Schaefer-400 regions. This generated a time-resolved phase coherence matrix (*N* × *N* dimensions, where *N* = 400) capturing moment-to-moment synchronization patterns. To reduce dimensionality while preserving dynamic network architecture, we implemented LEiDA (Koob and Volkow, 2016). For each temporal frame, the leading eigenvector (v1) of the phase coherence matrix was extracted, representing dominant synchronization topology through its sign-specific element values (positive/negative co-activation patterns). Each cluster centroid was back-projected to anatomical space using the Schaefer-Yeo template correspondence, revealing distinct RSNs configurations of each PL state. Next, we computed three dynamic indices: (1) occupancy (state prevalence across timepoints), (2) duration (consecutive state persistence), and (3) transition probabilities between states (computed by counting frame-to-frame transitions across the PL-state time series). For the transition probabilities between states, depending on the difference between the starting state and the destination state, transitions can be divided into inter-state transitions and self-state transition. Inter-state transitions refer to the switches between two different PL states (e.g., from PL-state 1 to PL-state 2), while self-state transitions indicate that the brain remains in the same PL state across adjacent timepoints (e.g., from PL-State 1 to PL-State 1). The self-transition probability serves as an indicator of temporal stability, reflecting how likely the brain is to remain in a given configuration without switching. A higher self-transition probability suggests greater neural inertia or “stickiness,” meaning that once the brain enters that state, it tends to persist longer—complementing other temporal metrics such as fractional occupancy and duration. Finally, statistical evaluation focused on identifying k-specific PL states showing significant longitudinal changes in temporal characteristics following rTMS intervention. This multi-step approach quantifies temporal reconfiguration of whole-brain network states, revealing stable connectivity patterns modulated by neuromodulation therapy. To evaluate the robustness of our approach, we assessed the consistency of brain states identified under different values of k in the k-means clustering. The corresponding figures and analyses have also been included in the Supplementary material.

### Statistical analysis

Demographic and clinical characteristics, including age, gender, educational attainment, monthly income, age of cocaine use onset, and total years of use, were tabulated for cohort characterization. Longitudinal changes in clinical scales (VAS, CCQ-G, HARS, and CCQ-N) were assessed via *t*-tests across three timepoints: baseline (pre-rTMS), 2-week post-intervention (post-rTMS-2 W), and 3-month follow-up (post-rTMS-3 M). PL state dynamics (occupancy, duration, and transition probabilities) were compared between pre-rTMS and post-rTMS-2 W periods using paired t-tests, with Benjamini–Hochberg false discovery rate (FDR) correction applied to all pairwise comparisons (*q* < 0.05). State metrics demonstrating significant longitudinal changes (pre-rTMS vs. post-rTMS-2 W) were subsequently entered into partial correlation analyses to examine monotonic associations with clinical symptom improvements measured at post-rTMS-2 W and post-rTMS-3 M, respectively. For each pair of interest, we computed Spearman partial correlations, statistically controlling for age, monthly income, and years of education as covariates. This approach allowed us to assess the direct association between dynamic brain state alterations and clinical outcome improvements, independent of potential demographic confounders. Analyses were restricted to PL state features showing FDR-corrected significance in prior group comparisons, ensuring hypothesis-driven inference. All statistical thresholds were set at FDR-adjusted *p* < 0.05.

## Results

### k-means optimal cluster number selection (*k* = 4)

**Figure 2:**
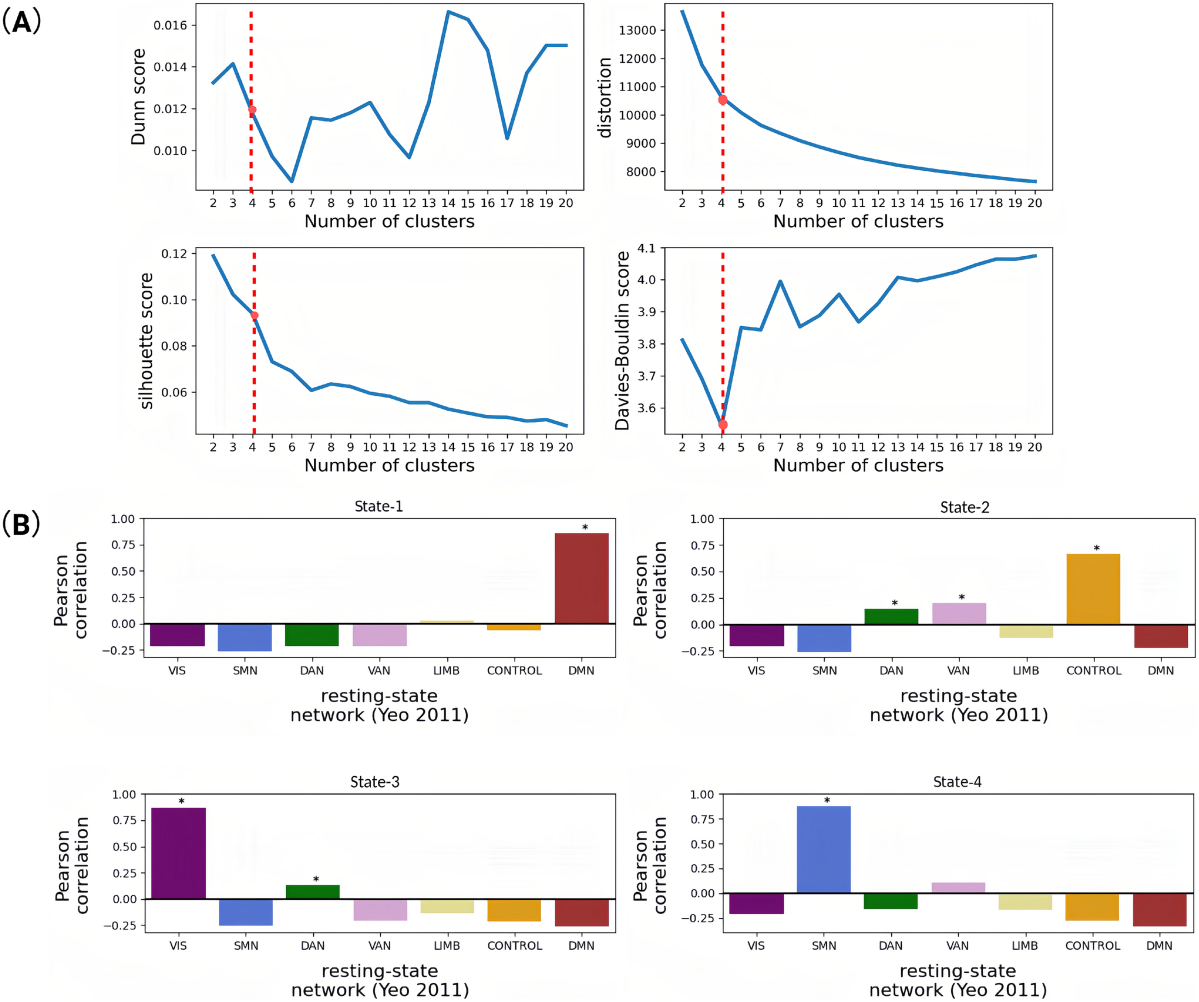
Correlation of brain functional networks obtained based on cluster analysis and indices for evaluating the quality of these clustering results. **(A)** Trend graph of four different clustering evaluation indicators changing with the number of clusters. When the number of clusters is 4, the Davis–Boulding index is low, which also indicates that the clustering effect is good. **(B)** Pearson correlation coefficients between the spatial pattern of each dynamic state (State 1–4) and the seven resting-state networks. It aims to functionally characterize the four dynamic brain states identified via k-means clustering by mapping each state’s spatial distribution to the canonical resting-state networks defined by Yeo. This approach infers the dominant functional systems associated with each state (e.g., whether a state reflects default mode, visual, or control-related activation patterns), thereby enhancing the interpretability of dynamic state transitions. Each bar reflects the spatial correlation between the mean PL vector of a state and a binary mask for a given network. High positive values suggest a strong spatial similarity between the brain state and that functional network (^*^p < 0.05).

The k-means clustering (cosine similarity metric) partitioned these eigenvectors into recurrent PL states across multiple cluster solutions (*k* = 2–20). Cluster count optimization (*k* = 4) was guided by four validation metrics: Dunn Index (maximized at *k* = 4), distortion score (elbow point at *k* = 4), Silhouette coefficient (peak = 0.38), and Davies–Bouldin index (minimum at *k* = 4) (Figure 2A). This four-state solution demonstrated neurobiological consistency with canonical resting-state networks while minimizing intra-state variance.

### The identified PL states

Our state-space analysis revealed four distinct metastable configurations with differential couplings to canonical functional networks (Figure 2B). PL-State 1 exhibited predominant coupling with the default mode network (DMN), reflecting its preferential involvement in internally-oriented cognition including autobiographical memory and self-referential processing. PL-State 2 demonstrated strongest association with the frontoparietal control network (FPCN), with secondary engagements of dorsal attention (DAN), and ventral attention (VAN) systems. PL-State 3 showed selective engagement of visual network (VIS) regions coordinated with dorsal attention circuitry, suggesting a role in visuospatial attention and perceptual integration. PL-State 4 was characterized by somatomotor network (SMN) synchronization coupled with ventral attention co-activation, potentially underlying sensorimotor processing during environmental monitoring.

### Temporal features of PL states

**Figure 3:**
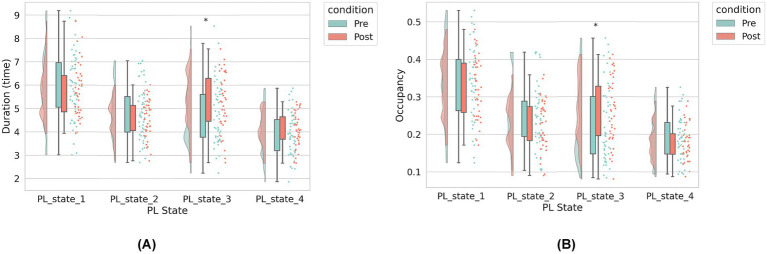
The dynamic brain state metrics under two conditions: pre-rTMS (before rTMS treatment) and post-rTMS-2 W (2 weeks after rTMS). **(A)** Left panel: Average duration (in seconds) of each PL state. **(B)** Right panel: Occupancy rate (i.e., proportion of total scanning time) for each PL state. The horizontal axis represents the four PL states identified via clustering. In both panels, green bars indicate pre-rTMS values, and orange bars indicate post-rTMS-2 W values. For each state, we compare pre- and post-rTMS metrics. Asterisks (*) denote statistically significant differences between conditions (^*^p < 0.05).

**Figure 4:**
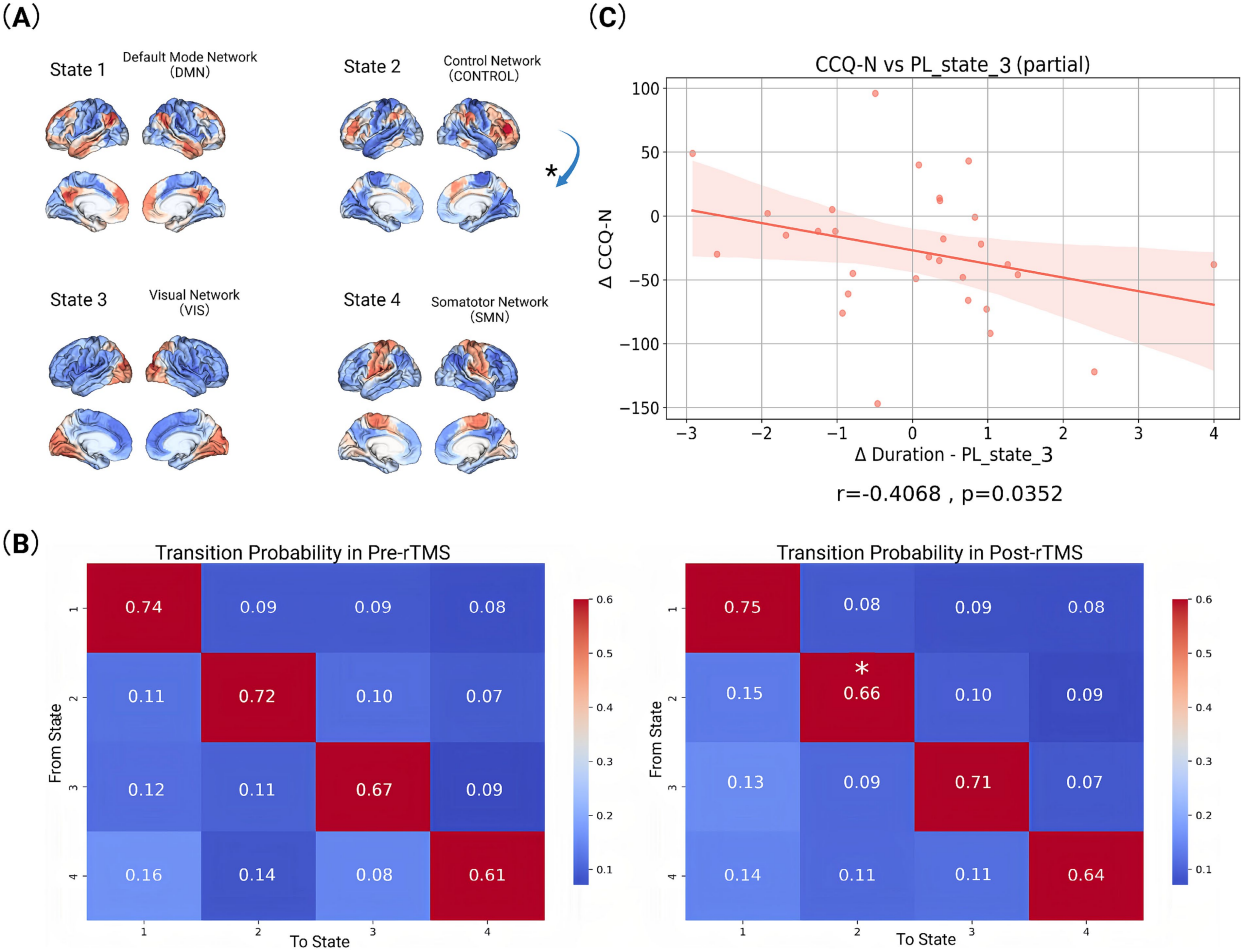
Visualized data and statistically analysed index. **(A)** Activation patterns of different functional brain networks in four states. The activation pattern in each state is represented by different colors, with red and orange representing higher activation levels and blue representing lower activation levels. Arrows mark the transition from one state to another (^*^p < 0.05). **(B)** State transition probabilities for baseline (pre-rTMS) and follow-up (post-rTMS-2 W). The values in each matrix represent the probability of transitioning from one state to another. The rows of the matrix represent the starting state and the columns represent the target state (^*^p < 0.05). **(C)** Correlation between changes in scale indicators and brain state indicators before (pre-rTMS) and after rTMS treatment (post-rTMS-2 W). Each plot includes a fitted line and confidence interval.

Longitudinal analysis of temporal dynamics across PL states revealed selective neuromodulatory effects following rTMS intervention (Figure 3). Notably, PL-State 3 (VIS-dominated) exhibited a significant increase in duration, for post-rTMS-2 W compared to pre-rTMS (*p* = 0.02). In contrast, PL-State 2 (FPCN-centric) and the SMN-dominated PL-State 4 showed minimal temporal alterations. Likewise, the DMN-centric PL-State 1 remained temporally stable across sessions. Longitudinal changes in metastable state transitions were also quantified through probabilistic modeling of phase-locking dynamics (Figures 4A,B). Our findings demonstrate that dlPFC-targeted rTMS elicited distinct alterations in dynamic brain states: (1) decreased self-transition probability of State 2 (frontoparietal control network-dominant) (*p* < 0.05), suggesting stabilization of cognitive control processes (see Figure 4B); (2) significantly increased dwell time and fractional occupancy of State 3 (visual network-dominant) (*p* < 0.05), indicating prolonged engagement of sensory processing systems (see Figure 4A).

### Clinical symptom modulation through PL state reconfiguration

While neuromodulation studies have established rTMS-induced changes in static connectivity patterns, the temporal dynamics linking network state transitions to symptom evolution remain poorly characterized. To address this mechanistic gap, we interrogated longitudinal relationships between dynamic phase-locking (PL) state reconfiguration and multi-domain clinical improvement. Longitudinal Spearman correlations revealed state-specific associations between dynamic network reconfiguration and symptom improvement (Figure 4C). At 2-week follow-up, decreased cocaine craving severity (CCQ-N) correlated with increased duration time of State 3 (*r* = −0.4068, *p* = 0.0352). No statistically significant correlations were observed between other PL-state metrics and clinical scale changes across assessment periods.

## Discussion

Our study elucidates the neuromodulatory effects of rTMS on dynamic brain network reorganization in CUD. Through LEiDA analysis, we demonstrate three key rTMS-induced effects: (1) decreased self-transition probability of State 2 (FPCN-dominant), indicating reduced metastability of cognitive control networks; (2) significantly increased dwell time and occupancy of State 3 (VIS-dominant); and (3) a robust negative correlation between State 3 dwell time and craving reduction (*r* = −0.4068, fp = 0.0352). These results collectively suggest a compensatory network reconfiguration mechanism wherein diminished FPCN stability permits enhanced sensory processing through VIS network engagement. We propose that reduced FPCN metastability reflects therapeutic disruption of maladaptive overcontrol patterns, while prolonged VIS states facilitate bottom-up reprocessing of craving stimuli through sensory extinction mechanisms (Torregrossa et al., 2011). The inverse VIS-craving relationship confirms this sensory-driven therapeutic action, positioning VIS state dynamics as a novel biomarker for rTMS efficacy in addiction treatment.

### Effects on brain networks

Our findings demonstrate that rTMS induces significant reorganization of dynamic brain states, particularly modulating VIS and CONTROL/FPCN networks. These alterations align with established models of addiction neuropathology, where impaired prefrontal regulation disrupts cognitive-emotional integration in substance use disorders (Koob and Volkow, 2016; Goldstein and Volkow, 2011). Crucially, we observed a significant increase in dwell time and fractional occupancy of the VIS-dominant state (State 3) post-rTMS, suggesting enhanced sensory network stabilization. This VIS-state prolongation was significantly negatively correlated with craving intensity (CCQ-N: *r* = −0.4086, *p* = 0.0352), indicating its direct relevance to therapeutic outcomes. We propose this reflects strengthened bottom-up sensory gating that attenuates drug-cue salience through perceptual desensitization. Concurrently, decreased self-transition probability in the CONTROL-dominant state (State 2) suggests reduced metastability of cognitive control networks. This decreased rigidity in top-down regulation may facilitate disengagement from compulsive craving patterns. These temporally specific dynamics extend prior static connectivity models (Dipasquale and Cercignani, 2016), revealing that rTMS reorganizes not only spatial network interactions but also the temporal architecture of functional states. Such metastable shifts may recalibrate prefrontal-executive hierarchies, counteracting the hyperstable network configurations characteristic of addiction.

### Differential network responsivity to dlPFC stimulation

Our temporal analysis reveals distinct neuroplasticity phases underlying rTMS efficacy: acute craving reduction at 2-week follow-up correlated significantly with destabilization of the visual network-dominant state (State 3), evidenced by increased dwell time, indicating early therapeutic effects involve disrupting sensory processing of craving cues. Besides, the selective increase in dwell time for State 3 (visual network; VIS) following dlPFC-targeted rTMS, contrasted with non-significant alterations in States 1 (DMN), 2 (FPCN), and 4 (SMN), may reflect network-specific responsiveness to neuromodulation. State 3 exhibited the highest metastability in our cohort, suggesting greater susceptibility to perturbation compared to more stable states. Methodologically, the LEiDA framework’s sensitivity to low-frequency, high-amplitude fluctuations characteristic of VIS activity may prioritize detection of visual network dynamics over subtler higher-order network changes. The absence of significant effects in other states does not negate their functional relevance but rather highlights the temporal specificity of acute rTMS effects, where VIS modulation may represent an early biomarker preceding longer-latency reorganization in cognitive networks.

### Shared neurobiological mechanisms

These network-level changes support rTMS as a transdiagnostic modulator restoring temporal coordination across functional systems. The VIS-state stabilization parallels Dipasquale and Cercignani’s (2016) findings of rTMS-enhanced sensory-limbic integration, suggesting conserved mechanisms for dampening pathological salience. Here, prolonged VIS engagement provides critical bottom-up recalibration of early perceptual circuits, establishing a stabilized sensory foundation for higher-order regulation. Meanwhile, decreased CONTROL-state self-transition probability reflects reduced cognitive rigidity—a finding echoing Zilverstand et al.’s (2018) framework linking PFC-striatal inflexibility to impaired behavioral control in CUD. The significant inverse VIS-craving relationship provides mechanistic validation: enhanced sensory network engagement directly suppresses subjective craving intensity. Collectively, these results indicate rTMS exerts therapeutic effects through three synergistic mechanisms: (1) stabilizing sensory networks to attenuate drug-cue hyperresponsiveness, (2) reducing CONTROL-network metastability to enable cognitive flexibility, and (3) facilitating inter-state transitions to restore dynamic network equilibrium. Rather than region-specific suppression, rTMS appears to recalibrate whole-brain metastability—positioning the dlPFC as a flexible hub that reconfigures temporal hierarchies across sensory and cognitive domains to disrupt craving maintenance.

### Comparison with healthy-control rTMS studies

Previous studies have shown that rTMS targeting the dlPFC can induce widespread reconfigurations of large-scale brain networks, including the SMN, VIS, FPCN, DMN, and limbic systems, even in healthy individuals (van Hattem et al., 2025; Singh et al., 2019). In our CUD cohort, we find comparable reconfiguration patterns, particularly involving the SMN, VIS, and FPCN, which suggests a common neuromodulatory mechanism of dlPFC-rTMS. However, in patients with cocaine use disorder, such reconfiguration likely serves to normalize rather than merely modulate. Thus, the observed shifts in brain-state dynamics in our study may reflect therapeutically relevant network rebalancing, aiding improvements in decision-making and reductions in impulsivity.

### Limitations and implications

Several constraints in this study warrant consideration. First, the ground truth of the estimated brain states with rapid fluctuations is largely inaccessible in human. This limitation raises questions about the specificity of LEiDA-derived metastable states to rTMS neuromodulation effects. While fMRI-based state dynamics may be contaminated by systemic noise sources (e.g., physiological artifacts, neurovascular coupling variability), cross-modality validation studies demonstrate conserved spatiotemporal patterns of metastable switching. Nevertheless, converging patterns of neural activity and dynamic state switching have been validated in cross-modality studies, irrespective of the signal’s origin. Second, the high dropout rate (23% dropout at 3-month follow-up) reduces power to detect delayed neuromodulatory effects, particularly in HARS-associated network reconfigurations showing later effect emergence. Besides, subsampling bootstrap implementation in LEiDA is not applicable due to the limitations of the LEiDA method. While subsampling bootstrap provides theoretical advantages for stability assessment, its implementation in LEiDA is confounded by cluster label non-identifiability and centroid matching ambiguity. We mitigated this through rigorous k-sensitivity validation by assessing the consistency of brain states identified under different values of *k* in the k-means clustering (see Supplementary material). Moreover, due to the limited number of female participants in the original dataset, our primary analysis focused exclusively on male subjects. Notably, we also conducted a parallel analysis including the female participants using the same methodology. In both analyses, the four identified brain states exhibited a high degree of spatial correspondence with the Yeo networks, and similar patterns of state dynamics were observed—specifically, a significant decrease in the self-transition probability of State 2 and a significant increase in both the fractional occupancy and duration of State 3. The only notable difference was an additional significant decrease in the transition probability from State 3 to State 4 in the mixed-gender sample. These findings suggest that the observed reconfiguration patterns are relatively robust across different sample compositions, and may not be strongly influenced by sex. However, given the small number of female participants, we cannot draw definitive conclusions regarding sex-related effects, which require further validation in larger and more gender-balanced cohorts. For transparency, the full results of the supplementary analysis including female participants have been provided in the Supplementary material.

## Conclusion

In conclusion, this study deepens previous research by systematically analyzing coherence matrix dynamics, state transition patterns, and brain imaging indicators to elucidate the neuroregulatory mechanisms of rTMS in treating CUD. By identifying specific alterations in brain’s metastable states and their correlation with clinical symptom improvement, our findings provide a mechanistic framework for how rTMS modulates addiction-related neural circuits. The insights of our results may contribute to the advancement of precision medicine by paving the way for individualized neuromodulation strategies based on dynamic brain networks.

## Data Availability

The datasets presented in this study can be found in online repositories. The names of the repository/repositories and accession number(s) can be found below: https://openneuro.org/datasets/ds003037/versions/2.1.0.
