# Supplementary material for: Dynamic brain network reconfiguration following rTMS in males with cocaine use disorder
Source: Front Hum Neurosci. 2025 Aug 21;19:1603888. doi: 10.3389/fnhum.2025.1603888 (PMC12408571; doi:10.3389/fnhum.2025.1603888)
Supplement: Supplementary file 1 [file Data_Sheet_1.pdf]

Figure S1. Consistency of LEiDA-inferred metastable states across various state clustering number.A. Cluster centroids of LEiDA-inferred eigenvectors corresponding to the metastable state that exhibited the most significant pre- vs post-treatment changes in both fractional occupancy and dwell time (specifically, State 3 at K=4) are visualized for a range of clustering solutions from K=2 to K=20. These state patterns demonstrate a consistent and stable network configuration across varying K. This suggests that the treatment-sensitive state identified at K=4 is not an isolated finding but reflects a robust dynamical pattern that re-emerges across different state resolutions.The continuous color bar represents the LEiDA eigenvector values for each brain region, indicating the degree of phase alignment within functional communities (red vs. blue). The discrete color bar below reflects the assignment of each region to one of the 7 canonical cortical networks defined by Yeo et al[1].B. Pearson correlations between the treatment-sensitive state (K=4, State 3) and its counterparts across K=2 to K=20.Further supporting the stability of this functional state across clustering scales.Abbreviations:VN = Visual Network; SMN = Somatomotor Network; DAN = Dorsal Attention Network; VAN = Ventral Attention Network; LMB = Limbic system; FPCN = Frontoparietal Control Network; DMN = Default Mode Network.

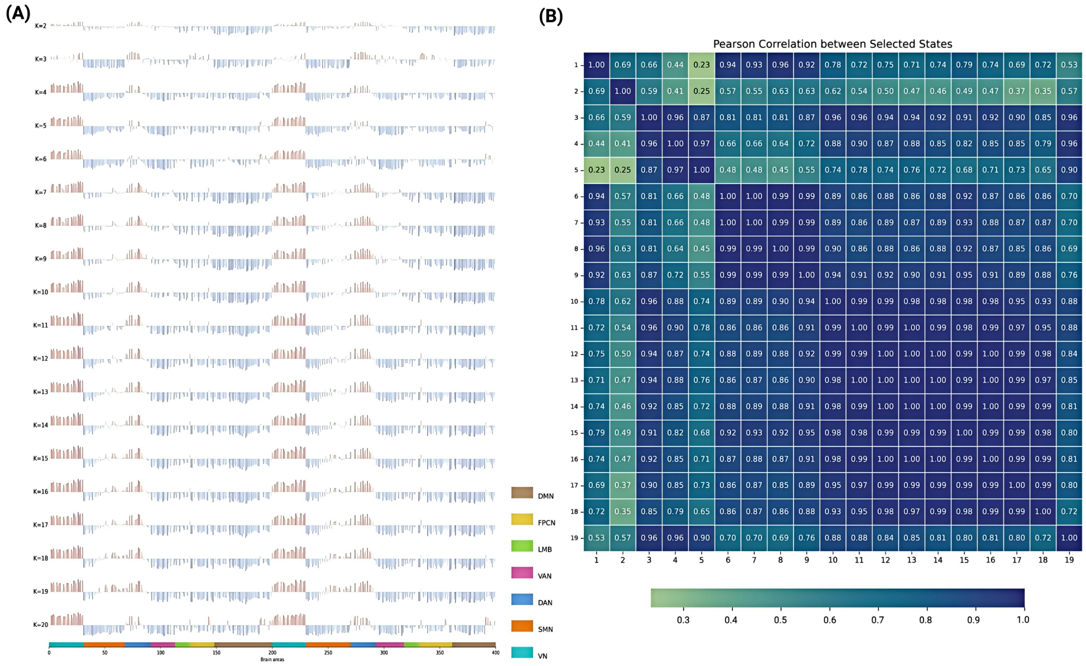

Figure S2. The comparison between a parallel analytical pipeline incorporating female participants to evaluate the robustness of our primary findings in the form of correlation of brain functional networks,duration time and occupancy,and transition probabilities. In both the male-only cohort and the mixed-sex cohort, the four recurrent brain states demonstrated high spatial correspondence with canonical Yeo functional networks. Critically, the core state dynamics exhibited consistent patterns of reorganization following rTMS: specifically, a significant reduction in self-transition probability for State 2 (frontoparietal control network; $p<0.01$ in both cohorts) and concurrent increases in both fractional occupancy ($p<0.01$) and mean duration

(p<0.05) for State 3 (visual network). The sole divergence emerged in transition dynamics: while the male-only cohort showed no significant change in transitions from State 3 to State 4), the mixed-sex cohort exhibited a marked decrease in this transition probability. This overall congruence indicates that the observed neuroplastic restructuring—particularly the stabilization of control networks and altered visual network dynamics—remains robust across varying sample compositions and is likely not strongly modulated by sex. However, the isolated difference in State 3→State 4 transitions suggests potential sex-dependent modulation of attentional network engagement, warranting targeted investigation in future studies with balanced cohorts.

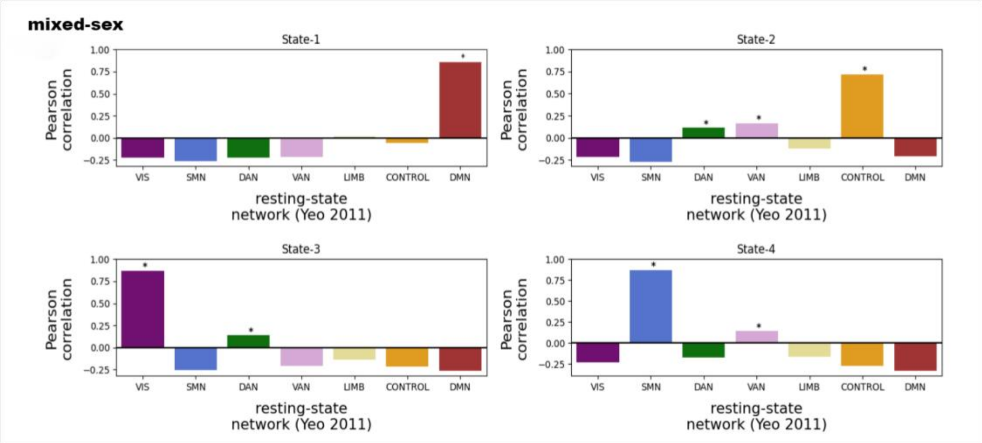

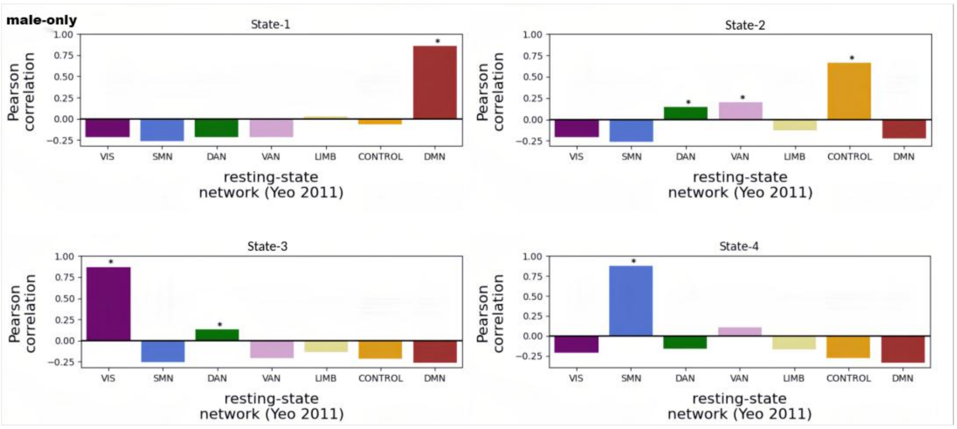

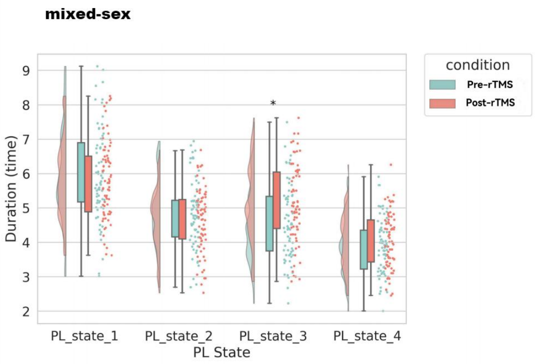
mixed-sex
condition
Pre-rTMS
Post-rTMS
Duration (time)
PL_state_1
PL_state_2
PL_state_3
PL_state_4
PL State
*

(a)

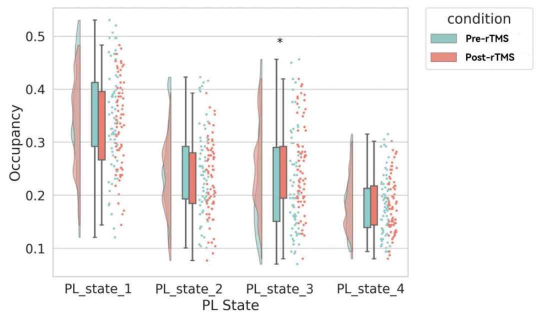
condition
Pre-rTMS
Post-rTMS
Occupancy
PL_state_1
PL_state_2
PL_state_3
PL_state_4
PL State
*

(b)

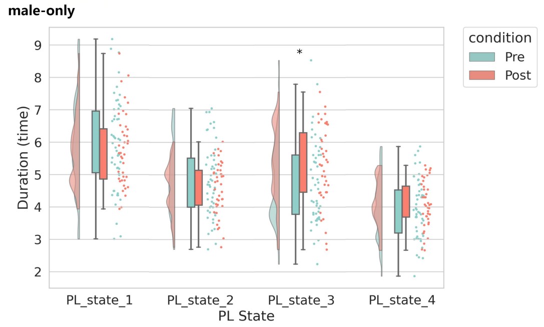
male-only
condition
Pre
Post
Duration (time)
PL_state_1
PL_state_2
PL_state_3
PL_state_4
PL State
*

(a)

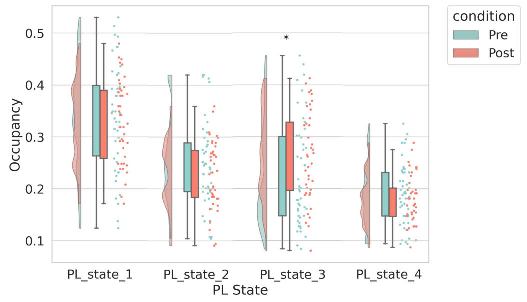
condition
Pre
Post
Occupancy
PL_state_1
PL_state_2
PL_state_3
PL_state_4
PL State
*

(b)

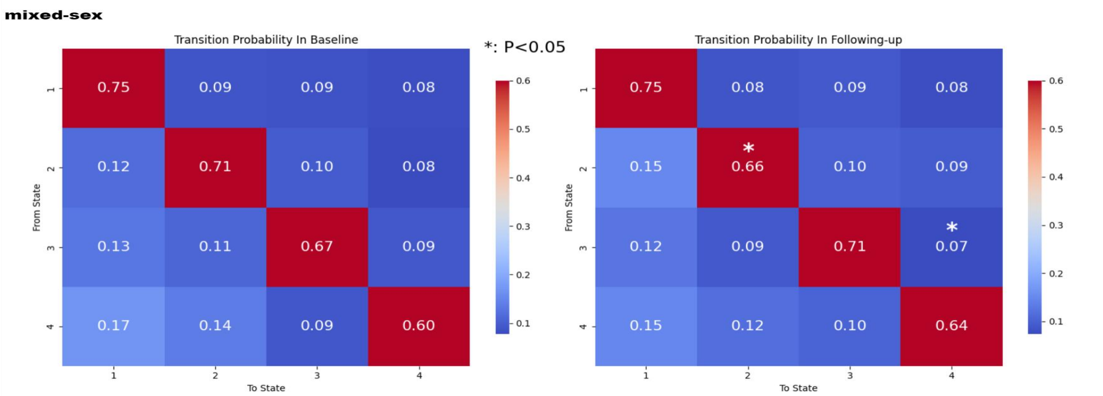
mixed-sex
Transition Probability In Baseline
*: P<0.05
Transition Probability In Following-up
From State
To State
0.75 0.09 0.09 0.08
0.12 0.71 0.10 0.08
0.13 0.11 0.67 0.09
0.17 0.14 0.09 0.60
0.75 0.08 0.09 0.08
0.15 0.66 0.10 0.09
0.12 0.09 0.71 0.07
0.15 0.12 0.10 0.64

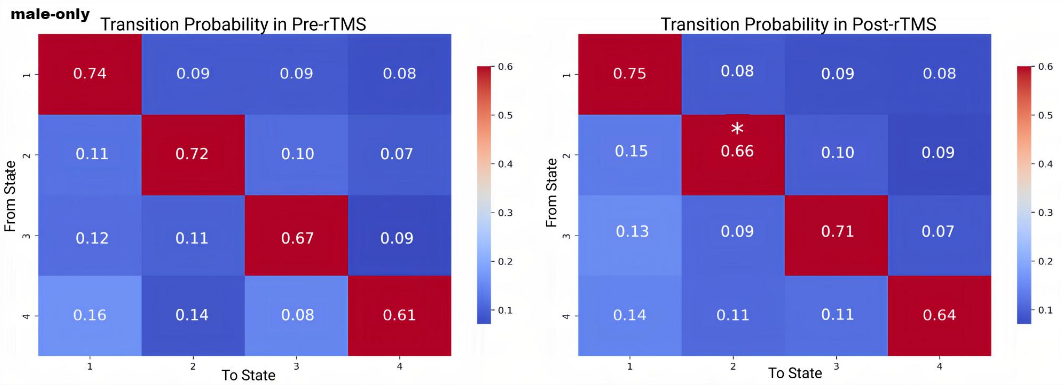
male-only
Transition Probability in Pre-rTMS
Transition Probability in Post-rTMS
From State
To State
0.74 0.09 0.09 0.08
0.11 0.72 0.10 0.07
0.12 0.11 0.67 0.09
0.16 0.14 0.08 0.61
0.75 0.08 0.09 0.08
0.15 0.66 0.10 0.09
0.13 0.09 0.71 0.07
0.14 0.11 0.11 0.64

References

1. Yeo BT, Krienen FM, Sepulcre J, Sabuncu MR, Lashkari D, Hollinshead M, Roffman JL, Smoller JW, Zöllei L, Polimeni JR, Fischl B, Liu H, Buckner RL. The organization of the human cerebral cortex estimated by intrinsic functional connectivity. J Neurophysiol. 2011 Sep;106(3):1125-65. doi: 10.1152/jn.00338.2011.
